# Supplementary material for: The effects of taxes on wealth inequality in Artificial Chemistry models of economic activity
Source: PLoS One. 2021 Aug 11;16(8):e0255719. doi: 10.1371/journal.pone.0255719 (PMC8357169; doi:10.1371/journal.pone.0255719)
Supplement: S1 Appendix — (PDF) [file pone.0255719.s001.pdf]

S1 Appendix. Gini coefficients for different scenarios. Fig 14 shows the Gini coefficients for the flat income tax regime of Section 5.1, while Fig 15 shows the Gini for a progressive income tax scenario (see Section 5.2).

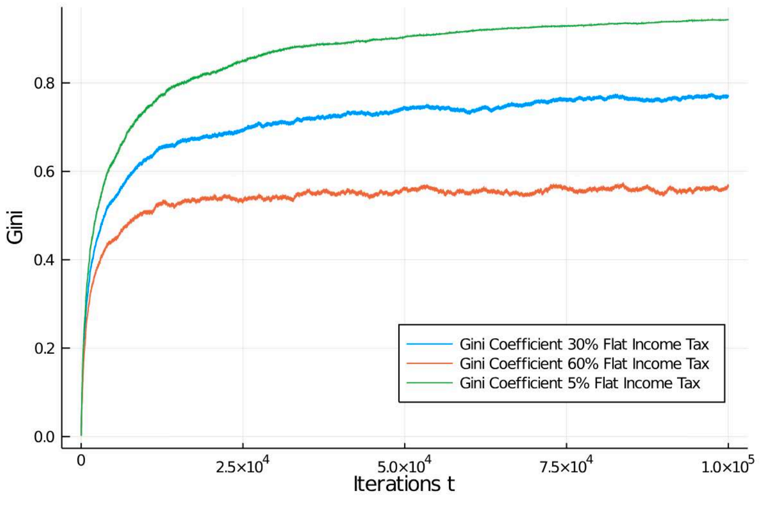

Fig 14. Comparison of Flat Income Taxes on Gini Coefficient. Gini coefficient for different flat income tax regimes at 30%, 60% and 5% with a population of 1,000 agents, starting from an equal distribution of wealth. Starting at "0", Gini indicates substantial inequality in all cases.

As is clearly visible from the application of income tax schemes at different levels, their effect on wealth distribution is much smaller than the natural pressure for unequal distribution of wealth from normal economic activity. What is striking is that a progressive income tax in the current model does not even have the same effect as a flat tax, likely due to the small spread in income distribution through individual economic transactions in our model.

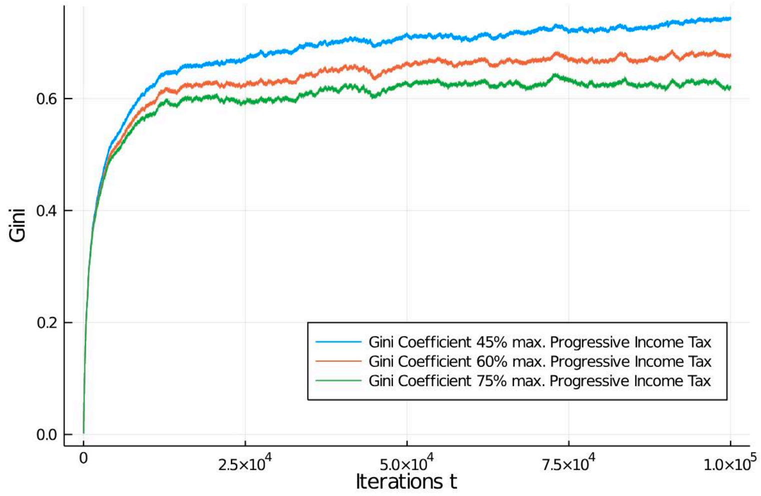

Fig 15. Comparison of Progressive Income Taxes on Gini Coefficient. Gini coefficient for different progressive income tax regimes with marginal tax rate of 45%, 60% and 75% with a population of 1,000 agents, starting from an equal distribution of wealth. Gini indicates even more substantial inequality than flat tax rate in all cases.

Turning finally to the effects of wealth taxes on economic inequality, we show Gini coefficients for the three flat wealth tax regimes we have considered in Section 5.3 (Fig 16). After quickly growing from ideal equality of $g = 0$, the Gini remains in a narrow band for all wealth tax regimes, with an average of around 0.27 for the lowest wealth tax. If one were to apply a 1% wealth tax only, though, the resulting wealth distribution would develop the classical exponential distribution, on par approximately with a 60% flat income tax.

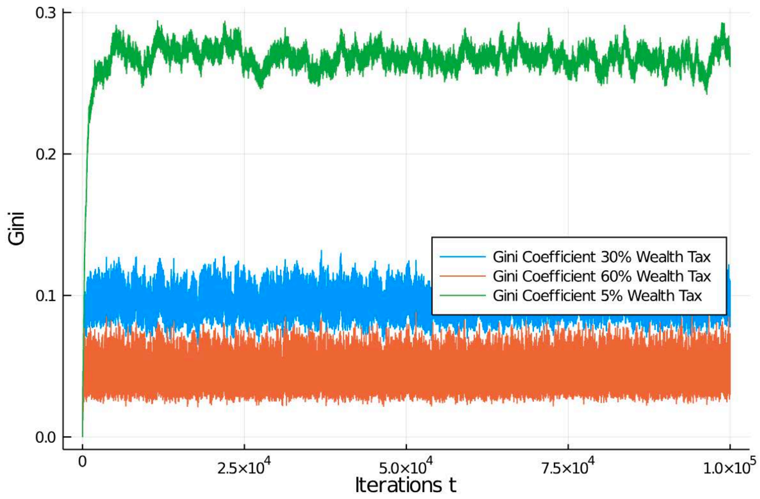

Fig 16. Comparison of Wealth Taxes on Gini Coefficient. Gini coefficient for different wealth tax regimes with a tax rate of 30%, 60% and 5% with a population of 1,000 agents, starting from an equal distribution of wealth. Gini indicates good economic equality, with fluctuations in narrow bands below $g = 0.3$.
