# Supplementary material for: The effects of taxes on wealth inequality in Artificial Chemistry models of economic activity
Source: PLoS One. 2021 Aug 11;16(8):e0255719. doi: 10.1371/journal.pone.0255719 (PMC8357169; doi:10.1371/journal.pone.0255719)
Supplement: S2 Appendix — (PDF) [file pone.0255719.s002.pdf]

S2 Appendix. Effective Tax Rates.

Effective tax rates are depicted in Fig 17 for a weak wealth tax regime. Effective tax rates take into account that generated tax amounts are re-distributed equally to the agent population after collection. Effective tax rates (after re-distribution) indicate that most taxpayers would effectively pay only a small amount of taxes as the system approaches an equilibrium state.

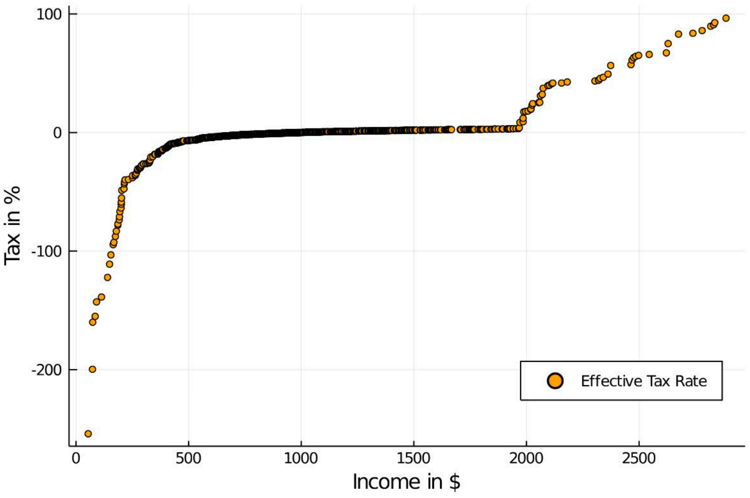

Fig 17. Effective Tax. Effective tax rates close to equilibrium ($t = 100,000$) for a weak wealth tax of 5%.
